# A bidirectional association between school environmental factors and physical activity habit formation among Chinese urban adolescents: a cross-lagged panel model

**DOI:** 10.3389/fpubh.2026.1775763

**Published:** 2026-03-24

**Authors:** Shengting Dai, Lu Dai, Yang Wu, Junlin Wang, Murui Ma, Jingfei Yan, Xinming Ye

**Affiliations:** 1 School of Sports Science and Engineering, East China University of Science and Technology, Shanghai, China; 2 Institute of Health Science and Engineering, East China University of Science and Technology, Shanghai, China; 3 Ministry of Physical Education, Shanghai Institute of Technology, Shanghai, China; 4 School of Physical Education and Health, East China Normal University, Shanghai, China

**Keywords:** adolescents, cross-lagged panel model, longitudinal study, physical activity habit formation, school environment

## Abstract

**Background:**

School environment is considered one of the important external factors that affect the formation of physical activity habits among teenagers, and its mechanism of action and time effect still require verification through longitudinal evidence.

**Methods:**

In this survey, junior and senior high school students in Shanghai were selected as samples. A three-time point tracking design was adopted to obtain valid questionnaires totaling 1,235. Descriptive statistics, correlation analysis, and cross-lag model analysis were conducted through SPSS 24.0 and Mplus 8.3, respectively, to explore the dynamic influence of school environmental factors on physical activity habits.

**Results:**

The results indicated that the attraction of facilities and environment, the norms of teachers' capabilities, and the guarantee of curriculum design could all be crucial positive predictors for the formation of physical activity habits at subsequent time points (*P* < 0.001), while existing activity habits of adolescents did not have a significant reverse impact on the above school factors. The model fits well: RMSEA = 0.042, CFI = 0.929, TLI = 0.868.

**Conclusion:**

School environmental factors, especially the guarantee mechanisms at the levels of teaching and curriculum, play a continuous promoting role in the formation of physical activity habits of teenagers. The study recommends that in reforming school physical education, more emphasis should be laid on integrating the curriculum to allow teacher support and the opening of sports venues in order to promote long-term consolidation and continuation of the activity habits.

## 1 Introduction

The World Health Organization (WHO) has included insufficient physical activity in adolescents as a global public health challenge. Research has shown that more than 80% of adolescents worldwide between the ages of 11 and 17 years do not achieve the recommended standard of at least 60 min of moderate-to-vigorous physical activity daily (1). Not getting enough exercise is also closely associated with chronic health issues in adolescents, such as obesity and cardiometabolic diseases, and has long-term effects on mental health and social adjustment in adolescence (2, 3). In recent years, accelerated social rhythms and increased digital media have resulted in an evident rise in sedentary behaviors among adolescents (4), while physical activity continues to show a downward trend, which has become an issue common to both developed and developing countries.

In China, the same problems are equally conspicuous. The results of large-scale national surveys reveal that a large percentage of children and adolescents in China do not meet the WHO's physical activity guidelines, with high amounts of screen time (5). This situation is intertwined with the increasing rates of overweight and obesity, high prevalence of myopia, and declining physical fitness, which are becoming a key factor affecting public health and educational quality. Many studies have confirmed the complex association between physical activity, screen time, and psychological symptoms like depression and anxiety (6, 7). To this end, the issue has already been given top priority at the national level. The “Healthy China 2030” Planning Outline proposes incorporating student physical health into the education quality evaluation system (8), while “Physical Education and Health Curriculum Standards for Compulsory Education (2022 edition)” further emphasizes the “Health First” educational philosophy, which requires schools to provide systematic support in curriculum design, staffing, and facility construction (9). Comparatively, both studies on the comparison of physical activity guideline across countries and analysis of the current national policy status in China equally point out that such healthy behavior should be promoted among adolescents under policy guidance (10, 11).

The school has been regarded as one of the most important living and learning environments for adolescents and one of the key settings for developing healthy lifestyles. Various factors influence students' opportunities for and patterns of physical activity, including the school's physical environment, structure of the curriculum, teachers' attitudes, interactions with their peers, and the school's culture. A systematic review published recently reported that offering adequate numbers of physical education classes, improving sports facilities, and creating a positive sports class atmosphere can largely improve the amount of physical activity students engage in daily (12). Research findings have shown that the school environment is strongly related to students' physical activity and sedentary behavior (13), and the opportunity for on-campus activity determines students' activity time (14). More specifically, the school physical and perceived environment, such as on-campus recess-time activity space, has been found to influence students' participation in activities accordingly (15). Meanwhile, students' physical activity habits are influenced by the school physical activity policy environment, class quality of physical education classes, class-level organizational factors, and teacher autonomy support by affecting their exercise self-efficacy, interest, physical literacy, and perceived barriers to exercise (16–19). Specialized scales regarding the impact of the school environment have also been developed and validated recently (18, 20–22). It has now become a key research hotspot to develop multi-faceted, school-based intervention programs or to explore new modes of intervention, such as education outside of the classroom, and mobile health applications (23–26).

However, most of the existing studies have focused on cross-sectional surveys that have, through correlational analysis, indicated associations between the school environment and physical activity but have failed to pay due attention to the long-term evolution of this relationship and its causal mechanism. The formation of physical activity habits is a dynamic developmental process among adolescents, and this may have longitudinal interactions with internalizing symptoms (for example, mental health) and learning engagement. Define “physical activity habit formation” narrowly as behavioral stability with increasing automaticity, rather than simply higher physical activity level or intention. Thus, our measure focuses on regular repetition and consistency over time and related self-regulatory features, aiming to capture habit-like persistence instead of single-wave participation. In order to deeply understand the dynamic relationships between these variables, there is an urgent need to introduce longitudinal research designs, particularly cross-lagged panel analysis. Currently, cross-lagged models have been successfully applied to explore the dynamic relationships between adolescent physical activity, screen time, and depressive symptoms, and between physical activity, sedentary time, and sleep, confirming that the method is effective for explaining complex behavioral causal chains. Nevertheless, in the specific domain of the long-term and dynamic impact of school environmental factors on the development of physical activity habits, such longitudinal studies remain rather few and far between. Scholars have pointed out that future studies should also attach more importance to the sustained effects of the school environment over time and explore how policy and educational interventions can offer support for the stable development of exercise behavior among students at different school stages.

This study adopts the Socio-ecological Model as its core theoretical framework, combined with Self-determination Theory, to establish a multi-level analytical perspective. As a key microsystem, the school involves physical facilities, teaching quality, peer interaction, and curriculum design. Through psychological mediators such as motivation and self-efficacy, it influences the formation and maintenance of physical activity habits among adolescents, clarifying the multi-layered pathways through which environmental factors shape health-related behaviors. This study will explore the dynamic mechanism of how school environmental factors influence the formation of physical activity habits among urban adolescents in China through a longitudinal research design. The plan is to implement multi-time point measurements among students from different regions and grades, focusing on the analysis of the pathways and temporal effects that external environmental variables—school facility conditions, curriculum setting, teacher guidance level, and campus sports atmosphere—play on the process of habit formation. It is expected that the research will disclose, through quantitative data, the intensity and direction of various environmental factors' influence on adolescent physical activity behavior, thus providing empirical support for school physical education curriculum design and campus environment optimization, which will help promote the sustainable development of physical activity and healthy growth among adolescents.

## 2 Research methods

### 2.1 Participants

This study employed a regional, stratified random sampling method, selecting Shanghai as the research setting. Three representative middle/high schools from the eastern (Pudong New Area), central (Xuhui District), and western (Songjiang District) regions were chosen as sample schools. In each school, one class from each grade was randomly selected as participants. The longitudinal survey was set at three measurement time points (T1–T3) with a 6-week interval between each, for a total period of 12 weeks. In the first survey (T1), 1,440 questionnaires were collected, with 1,389 valid responses. The second survey (T2) yielded 1,390 valid responses, and the third survey (T3) yielded 1,235. Ultimately, 1,235 complete three-wave tracking data sets were obtained. The sample consisted of 54.9% males and 45.1% females; 39.2% were from the eastern region, 33.9% from the central, and 26.9% from the western. The only-child ratio was 68.3%. The sample covered students from Grade 7 to Grade 12, demonstrating good representativeness and regional balance (based on Table 1). This study was approved by the Ethics Committee of East China Normal University (Approval No. HR 476-2020).

**Table 1:** Statistical description of participants' basic characters.

**Variable**	**Attribute**	**Frequency**	**Percent (%)**
Grade	Grade 7 (12–13 years old)	222	18.0%
	Grade 8 (13–14 years old)	178	14.4%
	Grade 9 (14–15 years old)	242	19.6%
	Grade 10 (15–16 years old)	172	13.9%
	Grade 11 (16–17 years old)	286	23.2%
	Grade 12 (17–18 years old)	135	10.9%
Sex	Male	642	51.9%
	Female	593	49.1%
	Eastern	484	39.2%
	Central	419	33.9%
	Western	332	26.9%
Only-child status	Yes	844	68.3%
	No	391	31.7%

### 2.2 Research instruments

The primary research instrument employed in this study is a questionnaire survey. (1) The Scale of Physical Activity Habit Formation Among Urban Chinese Adolescents was revised by the research team based on preliminary interviews and literature review, to evaluate adolescents' performance in physical activity participation, habit stability, and self-efficacy. The scale consists of 4 dimensions and 24 items, using a 5 point Likert scale. The internal consistency reliability (Cronbach's α) was 0.871. (2) The Scale of Influencing Factors on Physical Activity Habit Formation Among Urban Chinese Adolescents was developed to assess school environmental characteristics, covering four dimensions: Facility environment, teacher guidance, curriculum support, and campus atmosphere, with a total of 28 items also rated on a 5 point scale. Confirmatory factor analysis (CFA) demonstrated acceptable model fit (RMSEA = 0.048, CFI = 0.921), supporting adequate reliability and validity. Prior to formal distribution, the questionnaire was reviewed by experts and revised based on a pilot test to ensure item clarity, logical consistency, and developmental appropriateness for adolescent respondents.

### 2.3 Data processing methods

This study used SPSS 24.0 and Mplus 8.3 for statistical analysis. Descriptive statistics were first used to analyze the sample's basic characteristics and assess the reliability and validity of the instruments. Next, paired-samples *T*-tests, independent-samples *T*-tests, and repeated measures ANOVA were used to examine differences across gender, grade level, and region. Exploratory and confirmatory factor analyses (EFA and CFA) were conducted to validate the scale structures.

A cross-lagged model was then employed to analyze the causal relationship between school environmental factors and adolescent physical activity habits across different time points, revealing their bidirectional relationships and dynamic impact mechanisms. To address common method bias (CMB), Harman's single-factor test was used. The variance explained by the first factor in each of the three measurements was below 50%, indicating that CMB was not a serious concern in this study.

## 3 Research results

This section presents the main research results in three parts: first, the Pearson correlation analysis of school environmental factors and physical activity habit formation at three time points; second, the model fit indices of the cross-lagged panel model; third, the autoregressive effects and cross-lagged effects between variables, to explore the dynamic temporal relationship between the two variables.

### 3.1 Correlation analysis

To expose the underlying link between school environmental contextual factors and physical activities among adolescents, this study first applied Pearson correlation analysis to determine the correlation between Facility Environment Attraction (FEA), Standards of Teacher Competence (STC), Influence of Peer Relationship (IPR), Guarantee of Curriculum Setting (GCS), and Physical Activity Habit Formation (PAHF) at three different times (T1–T3). The correlation analysis uses a measurement approach that shows both the degree and direction of a linear relationship between a set of values, providing evidence to explore a lagged relationship and causal paths among the parameters under study, as indicated by the results presented in Table 2, whereby all parameters show a strong affirmative correlation at various times, signifying the high degree of interconnectedness between school environmental parameters and physical activities among adolescents.

**Table 2:** Correlation analysis results.

**Variable**	**M**	**SD**	**1**	**2**	**3**	**4**	**5**	**6**	**7**	**8**	**9**	**10**	**11**	**12**	**13**	**14**	**15**
1T1_FEA	4.086	0.865	1														
2T2_FEA	4.099	0.862	0.639^**^	1													
3T3_FEA	4.081	0.862	0.437^**^	0.568^**^	1												
4T1_STC	4.034	0.794	0.478^**^	0.297^**^	0.222^**^	1											
5T2_STC	4.025	0.799	0.283^**^	0.468^**^	0.272^**^	0.628^**^	1										
6T3_STC	4.003	0.800	0.217^**^	0.281^**^	0.481^**^	0.482^**^	0.613^**^	1									
7T1_IPR	3.942	0.900	0.521^**^	0.337^**^	0.256^**^	0.496^**^	0.316^**^	0.236^**^	1								
8T2_IPR	3.926	0.885	0.317^**^	0.527^**^	0.304^**^	0.314^**^	0.490^**^	0.285^**^	0.644^**^	1							
9T3_IPR	3.918	0.894	0.211^**^	0.288^**^	0.524^**^	0.225^**^	0.264^**^	0.458^**^	0.474^**^	0.571^**^	1						
10T1_GCS	3.891	0.904	0.486^**^	0.297^**^	0.201^**^	0.523^**^	0.310^**^	0.260^**^	0.546^**^	0.342^**^	0.262^**^	1					
11T2_GCS	3.882	0.898	0.287^**^	0.487^**^	0.260^**^	0.301^**^	0.512^**^	0.322^**^	0.330^**^	0.538^**^	0.328^**^	0.650^**^	1				
12T3_GCS	3.872	0.911	0.210^**^	0.296^**^	0.496^**^	0.238^**^	0.317^**^	0.501^**^	0.248^**^	0.319^**^	0.555^**^	0.486^**^	0.618^**^	1			
13T1_PAHF	3.765	0.518	0.177^**^	0.145^**^	0.056^*^	0.197^**^	0.168^**^	0.075^**^	0.228^**^	0.170^**^	0.083^**^	0.232^**^	0.196^**^	0.103^**^	1		
14T2_PAHF	3.755	0.526	0.321^**^	0.254^**^	0.159^**^	0.355^**^	0.271^**^	0.186^**^	0.315^**^	0.250^**^	0.150^**^	0.334^**^	0.271^**^	0.173^**^	0.419^**^	1	
15T3_PAHF	3.753	0.524	0.281^**^	0.390^**^	0.279^**^	0.296^**^	0.403^**^	0.292^**^	0.253^**^	0.390^**^	0.263^**^	0.281^**^	0.443^**^	0.335^**^	0.379^**^	0.569^**^	1

^*^P < 0.05; ^**^P < 0.01; FEA, Facility Environment Attraction; STC, Specification of Teachers' Competence; IPR, Influence of Peer Relationship; GCS, Guarantee of Curriculum Setting; PAHF, Physical Activity Habit Formation.

Based on Table 2, correlation coefficients for the five core factors across the three time points were all significantly positive. Correlations for Facility Environment Attraction across time ranged from 0.437 to 0.639. Correlations for Specification of Teachers' Competence ranged from 0.482 to 0.628. Correlations for Influence of Peer Relationship ranged from 0.474 to 0.644. Correlations for Guarantee of Curriculum Setting ranged from 0.486 to 0.650. Correlations for Physical Activity Habit Formation ranged from 0.379 to 0.569, and all were statistically significant.

Facility Environment Attraction and Physical Activity Habit Formation were positively correlated at all time points, with the correlation stronger at T2. Correlations between Specification of Teachers' Competence and habit formation increased gradually from T1 to T3. Correlations between Guarantee of Curriculum Setting and habit formation remained positive across all three waves and peaked at T3. Correlations between Influence of Peer Relationship and habit formation remained strong but declined slightly over time.

Moderate to strong positive intercorrelations were observed among environmental factors. Correlations between Facility Environment Attraction and Specification of Teachers' Competence, and between Facility Environment Attraction and Guarantee of Curriculum Setting, both exceeded 0.45. A strong positive correlation was also found between Influence of Peer Relationship and Specification of Teachers' Competence.

### 3.2 Lagged effect analysis

To further explore the dynamic evolutionary relationship between school environmental factors and adolescent physical activity habits, this study, building on the correlation analysis, used a cross-lagged model to verify the temporal effects and causal paths of Facility Environment Attraction (FEA), Specification of Teachers' Competence (STC), Influence of Peer Relationship (IPR), Guarantee of Curriculum Setting (GCS), and Physical Activity Habit Formation (PAHF). Autoregressive paths were included in the model to control for the stable influence of prior levels on later development, thereby more accurately revealing the time-lagged effects between variables.

It can be learned from Table 3 that the RMSEA value, SRMR value, CFI value, and TLI value of this model are 0.042, the SRMR value is 0.041, the CFI value is 0.929, and the TLI value is 0.868.

**Table 3:** Model fit indices.

**Fit**	**χ2**	**df**	**χ2/df**	**RMSEA**	**SRMR**	**CFI**	**TLI**
Model	703.232	51	13.789	0.042	0.041	0.929	0.868
Criteria			< 5	< 0.08	< 0.08	>0.9	>0.9

**Figure 1:**
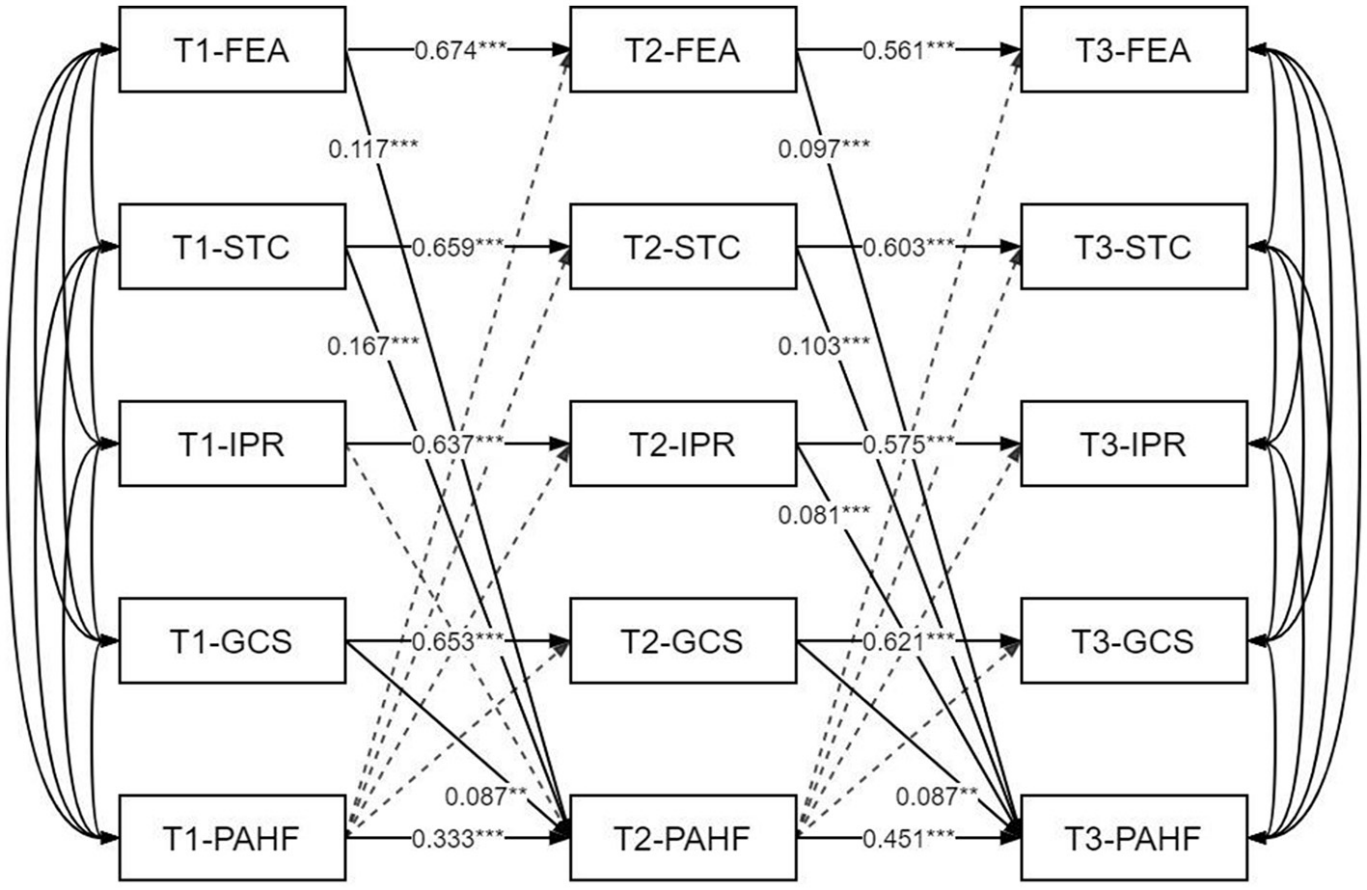
Cross-lagged model of the influence of school environmental factors on adolescent physical activity habit formation. FEA, Facility Environment Attraction; STC, Specification of Teachers' Competence; IPR, Influence of Peer Relationship; GCS, Guarantee of Curriculum Setting; PAHF, Physical Activity Habit Formation.

According to the results of model estimation, as shown in Figure 1 below, each major variable has a significant autoregressive effect before and after. The autoregressive coefficient of facility environment attraction was 0.674 from T1 to T2, and 0.561 from T2 to T3. Additionally, students' evaluations of the school's sports venues, equipment and usage experiences remained significantly consistent among the three time points. The two autoregressive coefficients of teachers' competency norms equaled 0.659 and 0.603, respectively (*P* < 0.001), Similarly, the influence of peer relationships (T1 → T2 = 0.637; T2 → T3 = 0.575) and the guarantee of curriculum Settings (T1 → T2 = 0.653; T2 → T3 = 0.621) reflects the continuity and sustainability of campus society and the teaching system. Lastly, the autoregressive coefficient for the formation of physical activity habits among teenagers are T1 → T2 = 0.333 and T2 → T3 = 0.451.

To further reveal the time series relationship between school environmental factors and the physical activity habits of adolescents, this study constructed a cross-lag model to test the autoregressive stability and lag effect of each variable. The model fitting results were good (χ^2^/df = 13.789, RMSEA = 0.042, SRMR = 0.041, CFI = 0.929, TLI = 0.868), indicating that the model structure was reasonable and could reflect the dynamic influence among variables.

As shown in Table 4, a cross-lagged path analysis explored the temporal stability of school environmental factors (FEA, STC, IPR, GCS) and their predictive effects on physical activity habit formation (PAHF), with data from three time points (T1–T3). Key results of autoregressive and lagged paths are summarized below (based on Table 4).

**Table 4:** Autoregressive and lagged path results for school environmental factors and physical activity habits.

**Path**	**Standardized coefficient**	***t***	***P***
T2_FEA← T1_FEA	0.674	26.64	0.000
T3_FEA← T2_FEA	0.561	20.80	0.000
T2_STC← T1_STC	0.659	26.03	0.000
T3_STC← T2_STC	0.603	24.32	0.000
T2_IPR← T1_IPR	0.637	22.62	0.000
T3_IPR← T2_IPR	0.575	22.47	0.000
T2_GCS← T1_GCS	0.653	25.27	0.000
T3_GCS← T2_GCS	0.621	23.97	0.000
T2_PAHF← T1_PAHF	0.333	9.08	0.000
T3_PAHF← T2_PAHF	0.451	13.07	0.000

FEA, Facility environment Attraction; STC, Specification of Teachers' Competence; IPR, Influence of Peer Relationship; GCS, Guarantee of Curriculum Setting; PAHF, Physical Activity Habit Formation.

All autoregressive paths from time 1 to time 2 and from time 2 to time 3 were statistically significant (*P* < 0.001). The strongest temporal stability was observed for FEA: T2 ← T1 (β = 0.674, *t* = 26.64); T3← T2 (β = 0.561, *t* = 20.80). STC also showed strong autoregression: T2← T1 (β = 0.659, *t* = 26.03); T3← T2 (β = 0.603, *t* = 24.32). IPR yielded β = 0.637, *t* = 22.62 (T2← T1) and β = 0.575, *t* = 22.47 (T3← T2). GCS showed β = 0.653, *t* = 25.27 (T2← T1) and β = 0.621, *t* = 23.97 (T3← T2). PAHF demonstrated smaller autoregressive effects: T2← T1 β = 0.333, *t* = 9.08; T3← T2 β = 0.451, *t* = 13.07. All coefficients were significant (*p* < 0.001). These results indicate substantial temporal stability across waves for the environmental and curricular factors, with habit formation showing moderate stability over time.

### 3.3 Comprehensive analysis of cross-lagged paths

To further reveal the longitudinal mechanism of school environmental factors on adolescent physical activity habits, this study conducted a comprehensive analysis of the key paths in the cross-lagged model, examining the direction and intensity of mutual influences between variables across different time periods.

As seen in Table 5, a cross-lagged path analysis was conducted to explore the cross-temporal predictive relationships between Facility Environment Attraction (FEA), Specification of Teachers' Competence (STC), Influence of Peer Relationship (IPR), Guarantee of Curriculum Setting (GCS) and Physical Activity Habit Formation (PAHF). Below are the key results of lagged path effects at different time points.

**Table 5:** Path coefficients of lagged effects from school environmental factors to physical activity habit formation.

**Lagged Path**	**Standardized Coefficient**	***t***	***P***
T2_PAHF← T1_FEA	0.117	3.819	0.000
T2_PAHF← T1_STC	0.167	5.516	0.000
T2_PAHF← T1_IPR	0.051	1.739	0.082
T2_PAHF← T1_GCS	0.087	2.666	0.008
T3_PAHF← T2_FEA	0.097	3.584	0.000
T3_PAHF← T2_STC	0.103	3.892	0.000
T3_PAHF← T2_IPR	0.081	3.374	0.001
T3_PAHF← T2_GCS	0.182	6.263	0.000

FEA, Facility environment Attraction; STC, Specification of Teachers' Competence; IPR, Influence of Peer Relationship; GCS, Guarantee of Curriculum Setting; PAHF, Physical Activity Habit Formation.

Predictive Effects of T1 Variables on T2_PAHF: Lagged path analysis from T1 to T2 showed differential predictive effects: T1_FEA (β = 0.117, *t* = 3.819, *p* < 0.001), T1_STC (β = 0.167, *t* = 5.516, *p* < 0.001) and T1_GCS (β = 0.087, *t* = 2.666, *p* = 0.008) significantly and positively predicted T2_PAHF. In contrast, T1_IPR had no significant predictive effect on T2_PAHF (β = 0.051, *t* = 1.739, *p* = 0.082), indicating no short-term stable impact of initial peer relationship influence on PAHF.

Predictive Effects of T2 Variables on T3_PAHF: All four T2 variables significantly predicted T3_PAHF: T2_FEA (β = 0.097, *t* = 3.584, *p* < 0.001), T2_STC (β = 0.103, *t* = 3.892, *p* < 0.001), T2_IPR (β = 0.081, *t* = 3.374, *p* = 0.001) and T2_GCS (β = 0.182, *t* = 6.263, *p* < 0.001). Notably, T2_IPR became significant (non-significant at T1–T2), reflecting a delayed impact of peer relationships, while T2_GCS showed the strongest predictive power.

## 4 Discussion

### 4.1 School provision and its influence

This study found that school facility environment attraction and Guarantee of Curriculum Setting had a stable positive effect on subsequent physical activity habits, which was consistent with international evidence on the role of school contextual provision in enhancing activity opportunities. For example, various country studies have identified that school playground space, recess activity zones, and extracurricular learning programs increase the level of physical activity during the school period. Wei et al. (27) concluded, in their review, that the school context offers an actionable strategic window both for reducing sedentary behavior and for promoting positive activity. Research in Brazil by Kuldas et al. (28) has also confirmed a significant association between the school environment and the activity and sitting times of adolescents. As indicated by “School Health Profiles” from the US CDC (29), there is a positive correlation between school-level policies, resource allocation, and student health behaviors. Further empirical research also shows that sustained school investment in sports resources and health education can improve adolescents' exercise behaviors and health status effectively. Kwon and Jang (30) pointed out that students in school sports safety education showed not only lower rates of injury but also stronger self-management in sports safety awareness and activity habits. Yang et al. (31) obtained through multilevel linear analysis that school-level dietary and exercise habit intervention was in line with a significant negative correlation with the level of blood pressure in students, indicating the synergistic effect between educational provision and lifestyle improvement. Li et al. (32) examined the long-term positive effect of regular exercise habits on the control of obesity in adolescents. It is indicated that the improvement of school provision promotes not only short-term student sports participation but also drives the sustainable formation and stable development of healthy behaviors through institutionalized educational interventions and environmental construction. The longitudinal result of this study further suggested that the cumulative effect of school provision can be scaled up across semesters and continuously exert a driving force for the formation of students' long-term physical activity habits. Hence, for resource investment and facility openness, schools should adhere to continuous semester-scaled improvements. For example, the appeal of the campus environment and curriculum guarantee of schools can be further enhanced by adding sports activity areas, improving equipment, opening facilities for use during extra-curricular hours, offering a variety of curriculum activities to create a comprehensive supply support mechanism of “institution-environment-behavior.”

### 4.2 The role of teacher support

The positive prediction of teacher competence and norms on subsequent physical activity habits in this study is consistent with the findings of many domestic and international studies. The pathway through which teacher-centered supportive teaching can enhance students' intrinsic motivation includes autonomy support, competence support, and relationship support. The scoping review by Christodoulakis et al. (33) indicated that systematic teacher training can significantly improve the activity level of adolescents in school; the longitudinal intervention study of Meerits et al. (34) also pointed out that the improvement of supportive behaviors of teachers can be transformed into increasing students' participation in after-school activities. Liu et al. (35) in an empirical evaluation of healthy physical education classrooms in China, pointed out that the reform of teaching organization and evaluation mechanisms will lead to higher student engagement and exercise enthusiasm. A longitudinal tracking study by Mikalsen et al. (36) showed that adolescents' activity levels have temporal stability, so continuous encouragement and support from the teacher are particularly important. Meanwhile, the indirect effect of teacher support on adolescents' psychological adjustment and wellbeing should not be ignored. The study by Fuentealba-Urra et al. (37) suggested that emotional self-regulation mediates the relationship between physical activity and subjective wellbeing, indicating that emotional support from teachers in the classroom can effectively promote students' mental health. The systematic review by Shao and Zhou (38) further indicated that the positive influence of teachers on students' exercise confidence, classroom feedback, and interactive atmosphere is an important social factor for adolescents to form lasting activity habits. Research by Fuentealba-Urra et al. (39). found that though sociodemographic characteristics may affect activity levels, the presence of teacher guidance could weaken this disparity significantly. This shows that teachers not only serve as the transmitters of sports knowledge but also as moderators of students' exercise behaviors and mental health; teaching support can promote the solid formation of the adolescent physical activity habit at the cognitive, emotional, and social level.

In line with the result of this study, it is recommended to further strengthen school-based development programs for teachers based on Shanghai's current reform of the physical education curriculum, integrating classroom management, teaching evaluation, and extracurricular extension activities into the same support system. Schools can establish communities of teacher learning, offer workshops on teaching physical education, and optimize incentive mechanisms to ensure teachers continuously provide support in both emotional and competence-based aspects. This will not only promote students' short-term participation in activities but also maintain the positive behavioral transmission across semesters, forming a virtuous cycle of health education ecology.

### 4.3 The role of peers and curriculum

This study showed that peer influence was not significant in the early period but showed a small positive effect in the subsequent period, which aligns with the explanation that peer effects may require time to accumulate. Zhou et al. (40) in a class-level study, found that peer interaction could slightly increase weekly physical activity time. Li et al. (41) based on analysis of national tracking data, pointed out that school atmosphere and peer ecology have a significant promotional effect on students' physical exercise levels. A systematic review by Contardo Ayala et al. (42) emphasized that composite strategies are more likely to trigger peer network effects than single interventions, especially when curriculum design is closely integrated with extracurricular activities. Meng et al. (43) reviewed the trajectory of China's curriculum reform, arguing that the “Health First” philosophy and the three-level curriculum management system provide a solid institutional foundation for schools to combine specialized and diversified physical education. Further research also corroborates the synergistic effect of curriculum and peer interaction from social-psychological and lifestyle perspectives. Fuentealba-Urra et al. (44) found that sociodemographic factors moderate the relationship between physical activity and subjective wellbeing, indicating that establishing an inclusive curriculum environment in diverse groups helps foster positive peer support. A two-year follow-up study by Takeda et al. (45) pointed out that good daily routines and curriculum arrangements can alleviate physiological interference with sports performance, demonstrating the supporting role of teaching pace and life regularity on activity persistence. Research by Taylor-Collins et al. (46) revealed that adolescents who engage in long-term helping or cooperative activities are more likely to form stable social action habits. This suggests that when school curricula integrate teamwork and social responsibility content, they can promote the long-term continuation and positive dissemination of physical activity habits within peer networks by strengthening group identity and emotional bonds. Combined with the sustained effect of guarantee of curriculum setting found in this study, it can be inferred that curriculum and peer factors have a synergistic effect on behavior formation. It is recommended that elements such as peer-assisted learning, group competition, interest-based clubs, and unit-based thematic learning be embedded into the curriculum system, with the aim of allowing students to cooperate and compete to build positive sports communities. Such a design would make use of classroom teaching and extracurricular activities in a coordinated manner, with mutual activation of the provision of curriculum and social support to enhance students' self-efficacy and sense of belonging during physical activity in group interactions and internalize their long-term habits.

### 4.4 Limitations of the study design

Cross-lagged panel model can reveal temporal predictive relationships between variables, but the results do not represent definitive causal effects due to the limitations of observational study design. The 6-week interval between the three measurement time points is relatively short for the study of “habit formation,” which is a long-term developmental process. Therefore, the results of this study are more applicable to the development and consolidation of physical activity habits in adolescents in the short term, and the long-term predictive effect needs to be verified by follow-up studies with longer time intervals.

This study only selected urban adolescents in Shanghai as the research object, and the results may not be directly generalized to rural adolescents in China or adolescents in other cultural contexts. The school environment in Shanghai is relatively well-resourced compared with rural areas, and the influence of school environmental factors on physical activity habits may vary with regional economic and educational development levels.

## 5 Conclusion

This study, based on three-time point longitudinal data and cross-lag models, examines the effect of school environment on the physical activity habits of urban adolescents. The results show that facility supply, teacher support and curriculum guarantee can stably predict subsequent habit improvement, while existing habits have no significant reverse effect on environmental factors. It is suggested that the opening of venues and the integration of courses be continuously optimized on a semester basis, teacher development be strengthened and peer assistance be embedded into the physical education curriculum system, so as to build a multi-dimensional school support environment for the formation of adolescents' physical activity habits. However, this study has several limitations: first, the 6-week interval between measurements is relatively short, which limits the interpretation of long-term habit formation processes; second, the sample only includes urban adolescents in Shanghai, restricting the generalizability to rural or other regional populations; third, self-reported scales may introduce subjective bias; finally, family and community environmental factors were not included, so the combined effects of multi-contextual influences remain unclear. Accordingly, future studies should adopt longer longitudinal intervals to examine the long-term effects of school environment on habit formation, recruit samples from urban, rural, eastern, central, and western regions to improve external validity, combine self-reports with objective devices (e.g., activity trackers) to reduce bias, construct multi-level models integrating school, family, and community contexts, and conduct intervention studies with randomized controlled designs to verify practical effectiveness.

## Data Availability

The raw data supporting the conclusions of this article will be made available by the authors, without undue reservation.

## 6 Appendices

The Chinese school environment influencing physical activity habits scale. This scale evaluates school environmental factors influencing adolescents' physical activity habits. The final validated scale includes 19 items across four dimensions. All items were rated on a 5-point Likert scale:

1 = Completely non-compliant

2 = Not very compliant

3 = Average

4 = Compliant

5 = Very compliant

**Table A1:**

**Factor**	**No**.	**Item**
Facility environment attraction (FEA)	FEA1	The centrally located sports facilities are more appealing to me for participating in physical activities
	FEA2	Convenient access to sports facilities is more enticing to me for participating in physical activities
	FEA3	The availability of sufficient sports facilities at school is more enticing to me for participating in physical activities
	FEA4	The school having a variety of facilities and equipment is more enticing to me for participating in physical activities
	FEA5	Effective management of school facilities and equipment is more enticing to me for participating in physical activities
Specification of teachers' competence(STC)	STC1	My teachers possess advanced teaching philosophies
	STC2	My teachers emphasize diverse reward methods
	STC3	My teachers only prioritize academic performance
	STC4	My teacher demonstrates technical movements in a standardized manner, ensuring thorough explanation and extensive practice
	STC5	My teacher can effectively organize us for sports activities or training
Influence of peer relationship (IPR)	IPR1	The fit physique of peers has a greater influence on my participation in physical activities
	IPR2	The peers' standard movement techniques have a greater influence on my participation in physical activities
	IPR3	My peers often participate in physical activities with me
	IPR4	My peers often encourage me to participate in physical activities
	IPR5	My peers are very concerned about my participation in physical activities
Guarantee of curriculum setting (GCS)	GCS1	Rich content in physical education courses can enhance my participation in physical activities
	GCS2	Academic exams leave me with no time for physical activities
	GCS3	Engaging in diverse sports club activities can enhance my participation in physical activities
	GCS4	Diverse sports club programs can enhance my participation in physical activities

## References

[1] Guthold R Stevens GA Riley LM Bull FC. Global trends in insufficient physical activity among adolescents: a pooled analysis of 298 population-based surveys with 1.6 million participants. Lancet Child Adolesc Health. (2020) 4:23–35. doi: 10.1016/S2352-4642(19)30323-2 31761562 PMC6919336

[2] Deng G Wen Y Cheng J Huang B Liu N. Analysis of the associations between moderate-to-vigorous physical activity and screen time on psychological symptoms among university students: a cross-sectional survey based on six geographic regions in China. BMC Psychiatry. (2024) 24:504. doi: 10.1186/s12888-024-05945-3 39014405 PMC11250946

[3] Xu Z Zhang F Su M Wang X. Physical activity and mental health in Chinese high school students: a cross-sectional study. Sci Rep. (2025) 15:9888. doi: 10.1038/s41598-025-94397-0 40121303 PMC11929784

[4] Amor-Barbosa M Ortega-Martinez A Carrasco-Uribarren A Bagur-Calafat MC. Active school-based interventions to interrupt prolonged sitting improve daily physical activity: a systematic review and meta-analysis. Int J Environ Res Public Health. (2022) 19:15409. doi: 10.3390/ijerph192215409 36430128 PMC9693257

[5] Guo M Zhu Y Wang X. Physical activity and recreational screen time among Chinese children and adolescents: a national cross-sectional study. Front Public Health. (2024) 12:1376330. doi: 10.3389/fpubh.2024.1376330 39050614 PMC11266031

[6] Chen S Jing L Li C Wang H. Exploring the nexus between moderate-to-vigorous physical activity, self-disclosure, social anxiety, and adolescent social avoidance: insights from a cross-sectional study in central China. Children. (2023) 11:56. doi: 10.3390/children11010056 38255369 PMC10814873

[7] Liu Y Zhang E Li H Ge X Hu F Cai Y . Physical activity, recreational screen time, and depressive symptoms among Chinese children and adolescents: a three-wave cross-lagged study during the COVID-19 pandemic. Child Adolesc Psychiatry Ment Health. (2024) 18:11. doi: 10.1186/s13034-024-00705-3 38243299 PMC10799442

[8] State Council of the People's Republic of China. Healthy China 2030 Planning Outline (2016). Available online at: https://www.who.int/teams/health-promotion/enhanced-wellbeing/ninth-global-conference/healthy-china#:~:text=Following%20the%20National%20Health%20Conference%2Cfounding%20of%20China%20in%201949 (Accessed February 15, 2026).

[9] Ministry of Education of the People's Republic of China. Physical education and health curriculum standards for compulsory education. J Shanghai Univ Sport. (2022) 46:1–9. doi: 10.16099/j.sus.2022.05.07.0004

[10] Hu D Zhou S Crowley-McHattan ZJ Liu Z. A comparative study of the physical activity guidelines for children and adolescents from five countries and WHO. Front Public Health. (2024) 12:1421843. doi: 10.3389/fpubh.2024.1421843 39071153 PMC11272551

[11] Chen S Hong J Milton K Klepac B Ma J Pedisic Z. Analysis of national physical activity and sedentary behaviour policies in China. BMC Public Health. (2023) 23:1024. doi: 10.1186/s12889-023-15865-8 37254122 PMC10230767

[12] Wang H Pang J Yang X Jia Y Huang X Yu L . School-based environment and physical activity in adolescents: a systematic review and meta-analysis. Prev Med. (2025) 191:108221. doi: 10.1016/j.ypmed.2025.108221 39765307

[13] Sales D da Silva Junior JP Bergamo RR de Oliveira LC Ferrari G Matsudo V. Association between school environment with sedentary behavior and physical activity intensity in children. Sci Rep. (2023) 13:6995. doi: 10.1038/s41598-023-33732-9 37117328 PMC10147915

[14] de Victo ER Ferrari G da Silva DRP Ferrero-Hernandez P Valenzuela C-F Sole D. Opportunities for physical activity in the school environment and their association with physical activity and sedentary behavior in Brazilian adolescents. Sci Rep. (2025) 15:9386. doi: 10.1038/s41598-025-94174-z 40102660 PMC11920261

[15] Wang Q Wang Y Zhou L Nan Y. Exploring the impact of school-based physical and perceived environments on students' physical activity during recess: a case study of four schools in Xi'an, China. Buildings. (2024) 14:3283. doi: 10.3390/buildings14103283

[16] Qin L Ho WKY Khoo S. The relationship between perceived quality physical education and 7-day physical activity among secondary school students in China: the mediating role of exercise self-efficacy. Arch Public Health. (2025) 83:242. doi: 10.1186/s13690-025-01731-z 41077573 PMC12516841

[17] Yin H Omar Dev RD Soh KG Zhang Y Tan Z Lian M. The relationship between school physical activity policy environment and physical literacy among primary school students: evidence from Henan Province, China. PLoS ONE. (2025) 20:e0330991. doi: 10.1371/journal.pone.0330991 41134756 PMC12551888

[18] Dai S Wu Y Yan J Zeng L. Development and validation of the Chinese school environment influencing physical activity habits scale. Front Educ. (2025) 10:1534843. doi: 10.3389/feduc.2025.1534843

[19] Zhao F Yang Z Xing L. The chain mediating role of interest and physical activity level in the PE teacher autonomy support to primary students' physical and mental health. Sci Rep. (2025) 15:37070. doi: 10.1038/s41598-025-21118-y 41131068 PMC12549878

[20] Zhou Y Wang L Chen R Wang B. Associations between class-level factors and student physical activity during physical education lessons in China. Int J Behav Nutr Phys Act. (2025) 22:1. doi: 10.1186/s12966-024-01703-6 39748427 PMC11697472

[21] Li M Wang Y Zan Z Lizhu L Lili Y. Physical literacy among Chinese elementary school students: the mediating role of physical knowledge and physical competency. BMC Public Health. (2025) 25:351. doi: 10.1186/s12889-025-21523-y 39875860 PMC11773747

[22] Li Q Li L He X Wang H. Exploring adolescent moderate-to-vigorous physical activity in China: mediating roles of school climate, perceived barriers, and physical education satisfaction. Risk Manag Healthc Policy. (2024) 17:3125–36. doi: 10.2147/RMHP.S497472 39687748 PMC11648554

[23] Elsborg P Mygind L Bølling M Klinker CD Melby PS Andreasen AH . Efficacy of education outside the classroom to increase adolescent physical activity. Sci Rep. (2024) 14:28213. doi: 10.1038/s41598-024-79138-z 39548298 PMC11568335

[24] Wang JW Zhu Z Shuling Z Fan J Jin Y Gao Z-L . Effectiveness of mHealth app–based interventions for increasing physical activity and improving physical fitness in children and adolescents: systematic review and meta-analysis. JMIR mHealth uHealth. (2024) 12:e51478. doi: 10.2196/51478 38687568 PMC11094610

[25] Terra LF Rezende LMT Ferreira Silva RM da Costa WP Minana-Signes V Noll M . Interventions on barriers to the participation of adolescents in physical activity: a systematic review. Int J Environ Res Public Health. (2025) 22:881. doi: 10.3390/ijerph22060881 40566312 PMC12193246

[26] Gasser M Nadenbousch AM Egger F Kamer M Valkanover S Schmidt M. Increasing adolescents' physical activity levels through a comprehensive school-based physical activity program: study protocol of the cluster randomized controlled trial active school. BMC Pediatr. (2024) 24:561. doi: 10.1186/s12887-024-05034-0 39232723 PMC11373237

[27] Wei B Jiang W Liu J Wu J Xu C Guo Y . Longitudinal relationships among early adolescent physical exercise, internalizing symptoms, and learning engagement: exploring within-person dynamics and the role of gender differences. Child Adolesc Psychiatry Ment Health. (2025) 19:104. doi: 10.1186/s13034-025-00965-7 41024128 PMC12482010

[28] Kuldas S Ranum BM Aspvik NP Wichstorm L Steinsbekk S. Physical activity, sedentary time, and sleep from childhood to young adulthood: a seven-wave cohort study of within-person relations. Sleep. (2025) 48:zsaf221. doi: 10.1093/sleep/zsaf221 40741983 PMC12696385

[29] Centers for Disease Control and Prevention. School Health Profiles 2020: Characteristics of Health Programs Among Secondary Schools (2022). Available online at: https://www.cdc.gov/school-health-profiles/media/pdf/cdc-profiles-2020.pdf (Accessed February 15, 2026).

[30] Kwon J Jang J. Impact of participation in sports safety education on sports injuries, sports safety awareness, and sports activity habits among young Korean athletes. Medicine. (2025) 104:e41589. doi: 10.1097/MD.0000000000041589 39993132 PMC11857000

[31] Yang Y Su H Chen Y Li T Ma L. Dietary and activity habits associated with hypertension in Kunming school-aged children and adolescents: a multilevel analysis of the study of hypertension risks in children and adolescents. Preventive Med Rep. (2024) 46:102854. doi: 10.1016/j.pmedr.2024.102854 39247205 PMC11378939

[32] Li H He W Liu G. Exercise habits and health behaviors on adolescent obesity. Acta Psychol. (2024) 245:104199. doi: 10.1016/j.actpsy.2024.104199 38490131

[33] Christodoulakis A Bouloukaki I Aravantinou-Karlatou A Margetaki K Zografakis-Sfakianakis M Tsiligianni I. The effectiveness of teaching the teacher interventions in improving the physical activity among adolescents in schools: a scoping review. Healthcare. (2024) 12:151. doi: 10.3390/healthcare12020151 38255040 PMC10815162

[34] Meerits PR Tilga H Koka A. Web-based intervention program to foster need-supportive behaviors in physical education teachers and parents: a cluster-randomized controlled study to increase students' intention and effort to engage in physical activity. BMC Public Health. (2025) 25:2142. doi: 10.1186/s12889-025-22590-x 40495137 PMC12150481

[35] Liu C Dong C Li X Huang H Wang Q. Analysis of physical education classroom teaching after implementation of the Chinese health physical education curriculum model: a video-based assessment. Behav Sci. (2023) 13:251. doi: 10.3390/bs13030251 36975276 PMC10045320

[36] Mikalsen HK Martins J Marques A Lagestad P. Longitudinal changes in adolescents' sedentary, light, moderate and vigorous physical activity levels. Educ Sci. (2024) 14:1193. doi: 10.3390/educsci14111193

[37] Fuentealba-Urra S Rubio A González-Carrasco M Oyanedel JC Cespedes-Carreno C. Mediation effect of emotional self-regulation in the relationship between physical activity and subjective well-being in Chilean adolescents. Sci Rep. (2023) 13:13386. doi: 10.1038/s41598-023-39843-7 37591897 PMC10435534

[38] Shao T Zhou X. Correlates of physical activity habits in adolescents: a systematic review. Front Physiol. (2023) 14:1131195. doi: 10.3389/fphys.2023.1131195 37179839 PMC10172932

[39] Fuentealba-Urra S Rubio A Flores-Rivera C Gonzalez-Carrasco M Oyanedel JC Castillo-Quezada H . Physical activity habits and their relationship with sociodemographic factors in Chilean adolescents. Front Psychol. (2022) 13:915314. doi: 10.3389/fpsyg.2022.915314 36059745 PMC9431025

[40] Zhou Z Li X Zhang Z. The peer effect in promoting physical activity among adolescents: evidence from the China education panel survey. Int Environ Res Public Health. (2023) 20:2480. doi: 10.3390/ijerph20032480 36767848 PMC9916313

[41] Li Z Xie S Chen W. The influence of environment on adolescents' physical exercise behavior based on family community and school micro-systems. Sci Rep. (2025) 15:12024. doi: 10.1038/s41598-025-91387-0 40199914 PMC11978958

[42] Contardo Ayala AM Parker K Mazzoli E Lander N Ridgers ND Timperio A . Effectiveness of intervention strategies to increase adolescents' physical activity and reduce sedentary time in secondary school settings, including factors related to implementation: a systematic review and meta-analysis. Sports Med Open. (2024) 10:25. doi: 10.1186/s40798-024-00688-7 38472550 PMC10933250

[43] Meng X Horrell A McMillan P Chai G. ‘Health First'and curriculum reform in China: the experiences of physical education teachers in one city. Eur Phys Educ Rev. (2021) 27:595–612. doi: 10.1177/1356336X20977886

[44] Fuentealba-Urra S Rubio-Rivera A González-Carrasco M Oyanedel JC Cespedes-Carreno C. The moderating role of sociodemographic factors in the relationship between physical activity and subjective well-being in Chilean children and adolescents. Int J Environ Re Public Health. (2021) 18:11190. doi: 10.3390/ijerph182111190 34769709 PMC8583316

[45] Takeda T Yoshimi K Imoto Y Shina M. Associations between sleep habits and interference of premenstrual symptoms in athletic performance in Japanese adolescent athletes: a cohort study over a 2-year period. Gynecol Endocrinol. (2020) 36:885–9. doi: 10.1080/09513590.2020.1734787 32124639

[46] Taylor-Collins E Harrison T Thoma SJ Moller F. A habit of social action: understanding the factors associated with adolescents who have made a habit of helping others. Voluntas. (2019) 30:98–114. doi: 10.1007/s11266-018-00070-8

